# Acute toxicity in comprehensive head and neck radiation for nasopharynx and paranasal sinus cancers: cohort comparison of 3D conformal proton therapy and intensity modulated radiation therapy

**DOI:** 10.1186/s13014-016-0600-3

**Published:** 2016-02-27

**Authors:** Mark W. McDonald, Yuan Liu, Michael G. Moore, Peter A. S. Johnstone

**Affiliations:** Department of Radiation Oncology, Winship Cancer Institute of Emory University, 1365 Clifton Rd NE Suite A1300, Atlanta, GA 30322 USA; Department of Biostatistics and Bioinformatics, Rollins School of Public Health of Emory University, Atlanta, GA USA; Department of Otolaryngology/Head and Neck Surgery, Indiana University School of Medicine, Indianapolis, IN USA; Department of Radiation Oncology, H. Lee Moffitt Cancer Center & Research Institute, Tampa, FL USA

**Keywords:** Proton therapy, Intensity modulated radiation therapy, Acute toxicity, Head and neck cancer

## Abstract

**Background:**

To evaluate acute toxicity endpoints in a cohort of patients receiving head and neck radiation with proton therapy or intensity modulated radiation therapy (IMRT).

**Methods:**

Forty patients received comprehensive head and neck radiation including bilateral cervical nodal radiation, given with or without chemotherapy, for tumors of the nasopharynx, nasal cavity or paranasal sinuses, any T stage, N0-2. Fourteen received comprehensive treatment with proton therapy, and 26 were treated with IMRT, either comprehensively or matched to proton therapy delivered to the primary tumor site. Toxicity endpoints assessed included g-tube dependence at the completion of radiation and at 3 months after radiation, opioid pain medication requirement compared to pretreatment normalized as equivalent morphine dose (EMD) at completion of treatment, and at 1 and 3 months after radiation.

**Results:**

In a multivariable model including confounding variables of concurrent chemotherapy and involved nodal disease, comprehensive head and neck radiation therapy using proton therapy was associated with a lower opioid pain requirement at the completion of radiation and a lower rate of gastrostomy tube dependence by the completion of radiation therapy and at 3 months after radiation compared to IMRT. Proton therapy was associated with statistically significant lower mean doses to the oral cavity, esophagus, larynx, and parotid glands. In subgroup analysis of 32 patients receiving concurrent chemotherapy, there was a statistically significant correlation with a greater opioid pain medication requirement at the completion of radiation and both increasing mean dose to the oral cavity and to the esophagus.

**Conclusions:**

Proton therapy was associated with significantly reduced radiation dose to assessed non-target normal tissues and a reduced rate of gastrostomy tube dependence and opioid pain medication requirements. This warrants further evaluation in larger studies, ideally with patient-reported toxicity outcomes and quality of life endpoints.

## Background

Head and neck radiotherapy is associated with significant acute and late toxicities including mucositis, taste perversion, dysphagia, odynophagia, weight loss, and xerostomia [1–3]. Improvements in radiation dose distribution, specifically the development of intensity modulated radiation therapy (IMRT), have improved the therapeutic ratio of treatment by reducing the incidence of toxicity through selective sparing of specific organs at risk, such as the parotid glands [4]. In multiple studies, more severe treatment toxicities have been correlated with increasing dose to regions such as the floor of mouth, oral cavity, submandibular glands, parotid glands, area postrema of the brainstem, and other sites [5–9], providing compelling evidence that optimized dose distributions translate into clinical toxicity reductions.

Proton therapy is a modality of radiation therapy distinguished from X-ray modalities by the Bragg peak, which allows the radiation to penetrate in to the depth of the target and then terminate, sparing normal tissues beyond the target from unnecessary radiation [10]. This has been hypothesized to improve the therapeutic ratio of treatment in a number of disease sites [11].

To quantify potential objective differences in acute toxicity, we reviewed our experience in treatment of patients with paranasal sinus and nasopharyngeal tumors receiving comprehensive head and neck radiation therapy using one of three techniques: IMRT, protons to the primary tumor site with concurrent matched IMRT to the neck, or comprehensive head and neck proton therapy alone.

## Methods

In this institution review board approved retrospective study, we evaluated patients treated between 2010 and 2014 at Indiana University Health University Hospital or affiliated sites in Indianapolis and the now-closed Indiana University Health Proton Therapy Center in Bloomington for primary malignancy of the nasopharynx, nasal cavity or paranasal sinuses, any T stage, N0-2 receiving radiation either definitively or following surgery, given with or without chemotherapy, who received radiation to the primary tumor site and bilateral cervical lymph node regions. Patients with a prior history of head and neck radiation or with a second concomitant active malignancy were excluded.

Between 2010 and 2014, 12 patients received comprehensive head and neck radiation with IMRT in Indianapolis. These patients did not receive proton therapy because either the patient faced logistical difficulties in arranging for daily travel or temporary relocation to our geographically distant proton treatment site (*n* = 8), or the attending physician did not discuss proton therapy as a treatment option (*n* = 4).

Between March 2010 and November 2012, 14 patients were treated in Bloomington with proton therapy to the primary tumor site with concurrent matched photon therapy to bilateral cervical lymph node regions, as previously reported in other experiences [12–14]. IMRT was used to treat the cervical lymphatics, with a half-beam block at the matchline. Two matchline positions were placed at the inferior extent of the clivus 0.5 cm apart, and setup altered between the two matchlines every day. The rationale in utilizing proton therapy to the primary tumor site was related to the proximity of the primary tumor or tumor bed to critical normal structures, and matched photon therapy was utilized for nodal irradiation consistent with other institutional experiences [12–14] and based on a belief that comprehensive nodal irradiation with proton therapy would be too time consuming or associated with minimal dosimetric gain. Our subjective experience was that acute toxicity was similar to patients treated with IMRT, and the practice of concurrent matched modalities too complex, requiring substantial additional resources including quality assurance.

In December 2012, in an effort to streamline patient care and potentially reduce toxicities, we developed a technique for comprehensive head and neck radiation using only proton therapy and implemented that in our practice, treating 14 patients meeting study inclusion criteria between 2012 and 2014. This technique has previously been described in detail [15]. With the implementation of comprehensive proton therapy, we did not treat with matched IMRT again.

Patient and treatment factors collected were age, gender, T and N stage, Karnofsky performance status prior to treatment, treatment modality, indication for radiation (definitive or adjuvant), incorporation of a neck dissection, prescribed radiation dose to the neck and to the primary tumor site, utilization of chemotherapy, type of chemotherapy, placement of a gastrostomy tube (g-tube) during treatment, whether the patient became g-tube dependent (defined as no more than sips of water by mouth) by the completion of treatment, whether they were g-tube dependent 3 months following completion of treatment, patient weight prior to initiation of treatment and each week during radiation, as well as at 1 and 3 months after completion of radiation, and opioid pain requirements. Opioid pain medication usage was converted to a daily equivalent morphine dose (EMD) [16]. The patient’s EMD prior to initiation of radiation served as the baseline and changes in the opioid medication usage were recorded each week during radiation, and at 1 and 3 months following completion of therapy. Due to variable documentation by different treating physicians, provider-assessed toxicity assessments were not uniformly available.

Our institutional approach for head and neck patients has been to recommend upfront placement of gastrostomy tubes, particularly for those receiving concurrent chemotherapy, although some patients decline and prefer a reactive approach. With the development of comprehensive proton therapy at the proton therapy center, the improved radiation dosimetry to the oral cavity, esophagus and larynx prompted a shift to a generally reactive policy of gastrostomy tube placement for those who demonstrated a clinical need. One proton patient had a gastrostomy tube placed prior to radiotherapy at the time of surgical resection with free flap reconstruction. One proton patient with esthesioneuroblastoma and bilateral retropharyngeal nodal involvement had a prophylactic gastrostomy tube placed due to concern for toxicity related to delivery of concurrent cisplatin and etoposide [17]. None of the patients treated with comprehensive proton therapy required placement of a gastrostomy tube during radiotherapy. All patients were evaluated by a dietician during treatment and provided with supplemental and meal replacement nutritional shakes at no cost.

Dosimetric information was exported from individual treatment plans for the following organs at risk (OARs): the esophagus (contoured from the cricoid to the caudal aspect of the aortic arch), the larynx (inclusive of the thyroid cartilage and postcricoid space abutting the vertebral body), the parotid glands (separated as the “better-spared” and “lesser-spared” glands), and the “oral cavity”, an avoidance structures whose lateral and anterior border was defined by the mandible, cranial by the maxillary bone and inclusive of any air cavity above the oral tongue, inferior including the floor of mouth, and posterior including the base of tongue. In some patients, regional nodal involvement or primary tumor extent precluded any ipsilateral parotid gland sparing, so not every patient had a “lesser-spared” parotid gland.

For the purpose of this study, patients were grouped according to the radiation modality used to treat the cervical lymphatics, so that those treated with comprehensive IMRT and those treated with IMRT to the neck matched to proton therapy to the primary site were combined for comparison against comprehensive proton therapy. To assess the validity of this grouping, we evaluated the mean dose to assessed OARs for patients receiving IMRT either alone or matched to proton therapy to the primary tumor site (Table 1). For patients with node negative disease, those treated with matched IMRT had reduced oral cavity radiation dose compared to those treated with comprehensive IMRT (mean 28.3 versus 44.4 Gy, *P* = 0.036). There was no statistically significant difference in mean dose to other assessed OARs.

**Table 1:** Comparison of mean dose to organs at risk for IMRT delivered alone or matched to proton therapy to the primary tumor site

	Node negative			Node positive		
Organ at risk	Alone (*n* = 2)	Matched (*n* = 8)	*P*	Alone (*n* = 10)	Matched (*n* = 6)	*P*
Oral Cavity	44.4	28.3	0.036	47.6	39.7	0.093
Larynx	46.3	38.9	0.117	43.5	46.2	0.492
Esophagus	26.0	29.4	0.296	35.6	36.8	0.562
Better spared parotid	31.6	26.5	0.192	32.9	35.3	>0.99
Lesser spared parotid	29.5 (*n* = 1)	28.8 (*n* = 8)	N/A	35.7 (*n* = 3)	37.2 (*n* = 3)	>0.99

*IMRT* intensity modulated radiation therapy, radiation dose expressed in Gy

Statistical analysis was conducted using SAS version 9.3 (Cary, NC). The significance level was set at 0.05. Descriptive statistics for each variable were reported. For the two cohorts of proton and IMRT nodal irradiation, univariate association of categorical variables was assessed with the Fisher exact test, and the Mann-Whitney U test for numerical covariates. The use of concurrent chemotherapy and the presence of cervical nodal disease (N+) were considered as clinically important confounders for inclusion in multivariable analysis of toxicity outcomes. The adjusted association between treatment modality and each toxicity outcome was estimated by exact logistic regression and linear regression for binary and numerical outcome respectively. The normality assumption was checked by residual plots. Due to a large number of patients with zero opioid pain medication requirements over time, the model assumptions of linear regression were violated, prompting alternate binary analysis using categories of opioid pain medication greater than baseline or the same or less than baseline requirement.

A subset analysis was performed of patients receiving concurrent chemotherapy in which Spearman’s rank correlation coefficient was used to assess for correlation between assessed continuous dosimetric variables and toxicity endpoints.

## Results

Forty patients met the inclusion criteria for this study. Table 2 reviews patient characteristics and treatment details, comparing those who received neck irradiation with IMRT and those with comprehensive irradiation using only proton therapy. There were imbalances between the two groups in several areas, with the proton cohort having more paranasal sinus primaries and a greater use of induction chemotherapy. Although not statistically significant in this sample size, the proton cohort had a greater percentage of T4 and N0 tumors.

**Table 2:** Patient and treatment characteristics

Variable	IMRT neck (*n* = 26)	Proton neck (*n* = 14)	*P*	Comparison
Median age (range)	54.1 (22–77)	46.7 (16–71)	0.98	
Gender				
Male	14 (53.8 %)	11 (78.6 %)	0.18	
Female	12 (46.2 %)	3 (21.4 %)		
Primary tumor site				
Nasopharynx	15 (57.7 %)	2 (14.3 %)	0.02	
Nasal/Paranasal	11 (42.3 %)	12 (85.7 %)		
Tumor Histology				
SCC	13 (50.0 %)	3 (21.4 %)	0.19	SCC vs non-SCC
Poorly differentiated carcinoma	5 (19.2 %)	0		
Sinonasal undifferentiated	4 (15.4 %)	5 (35.7 %)		
Esthesioneuroblastoma	1 (3.8 %)	5 (35.7 %)		
Neuroendocrine carcinoma	1 (3.8 %)	0		
Lymphoepithelioma	1 (3.8 %)	1 (7.1 %)		
High grade mucoepidermoid carcinoma	1 (3.8 %)	0		
KPS before radiation				
90–100	13 (50 %)	9 (64.3 %)	0.51	≥90 vs <90
80	9 (34.6 %)	3 (21.4 %)		
70	4 (15.4 %)	2 (14.3 %)		
T stage				
T4	13 (50 %)	11 (78.6 %)	0.10	T4 vs < T4
T3	8 (30.8 %)	2 (14.3 %)		
T2	4 (15.4 %)	1 (7.1 %)		
T1	1 (3.8 %)	0		
N Stage				
N0	10 (38.5 %)	10 (71.4 %)	0.09	N0 vs N+
N1	5 (19.2 %)	1 (7.1 %)		
N2	11 (42.3 %)	3 (21.4 %)		
Neck dissection				
Upfront	0	1 (7.1 %)	0.54	none vs other
None	25 (96.2 %)	12 (85.7 %)		
Adjuvant/Salvage	1 (3.8 %)	1 (7.1 %)		
Chemotherapy sequencing				
None	3 (11.5 %)	2 (14.3 %)	0.10	concurrent vs other
Induction	0	3 (21.4 %)		
Concurrent	23 (88.5 %)	7 (50.0 %)		
Concurrent and adjuvant	0	2 (14.2 %)		
Chemotherapy Type				
Cisplatin	15 (57.7 %)	6 (42.9 %)	0.14	cisplatin vs non-cisplatin
Cisplatin and etoposide	1 (3.8 %)	5 (35.7 %)		
Cisplatin, docetaxel, fluorouracil	2 (7.7 %)	1 (7.1 %)		
Carboplatin	2 (7.7 %)	0		
Carboplatin + taxol	1 (3.8 %)	0		
Cetuximab	2 (7.7 %)	0		
Smoking history				
Never	11 (42.3 %)	10 (71.4 %)	0.18	never vs any history
< 10 pack years	1 (3.8 %)	0		
> 10 pack years	14 (53.8 %)	4 (28.6 %)		
Active smoking at time of radiation	6 (23.1 %)	2 (14.3 %)	0.69	
Diabetes Mellitus	4 (15.4 %)	0	0.28	
Gastrostomy tube placement	22 (84.6 %)	2 (14.3 %)	<0.001	
Median primary tumor dose	71.8 Gy	71.4 Gy (RBE)	0.86	
Range primary dose	66–76.4 Gy	63–75.6 Gy (RBE)		
Median neck dose, node negative	52.3 Gy	50.2 Gy (RBE)	0.58	
Range neck dose, node negative	40.0–59.4 Gy	45.0–58.0 Gy (RBE)		
Median neck dose, node positive	68.3 Gy	72.9 Gy (RBE)	0.06	
Range neck dose, node positive	59.4–70.29 Gy	70.0–75.6 Gy (RBE)		

*SCC* squamous cell carcinoma, *vs* versus, *KPS* Karnofsky performance status, *RBE* relative biological effectiveness

**Fig. 1:**
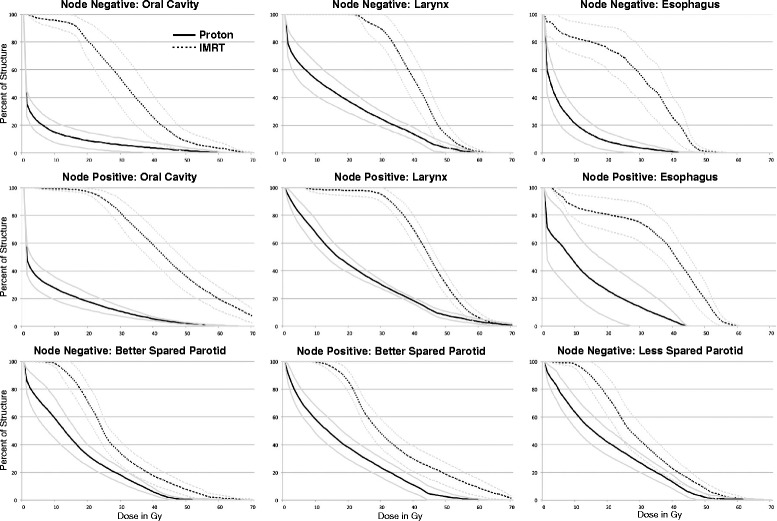
Mean dose volume histograms (DVHs) to organs at risk by radiation treatment modality, separated by node negative and node positive patients. The mean DVH is presented with 95 % confidence intervals

Figure 1 shows mean dose volume histograms for the assessed OARs by treatment modality, with 95 % confidence intervals, separated by patients treated for N0 versus N+ disease. Table 3 reports comparisons of the mean of the mean dose to OARs by treatment modality, separated for patients with N0 versus N+ disease. For patients with node positive disease, regional nodal disease precluded sparing of one parotid gland in 3 of 4 proton patients and 10 of 16 IMRT patients, precluding statistical dosimetric comparison of the “lesser-spared” parotid in the node positive subgroup. Table 4 reviews the association of treatment modality with toxicity outcomes in univariate analysis.

**Table 3:** Comparison of mean dose to organs at risk by radiation modality when IMRT or proton therapy was used for treatment of the cervical lymph nodes

	Node negative			Node positive		
Organ at risk	IMRT (*n* = 10)	Proton (*n* = 10)	*P*	IMRT (*n* = 16)	Proton (*n* = 4)	*P*
Oral cavity	31.5	5.1	<0.001	44.6	8.5	<0.001
Larynx	40.4	16.8	<0.001	44.5	21.4	0.002
Esophagus	28.7	5.9	<0.001	36.0	12.1	<0.001
Better spared parotid	27.5	15.4	<0.001	33.8	17.4	0.001
Lesser spared parotid	29.5 (*n* = 9)	19.0 (*n* = 10)	0.006	35.5 (*n* = 6)	18.4 (*n* = 1)	N/A

*IMRT* intensity modulated radiation therapy, Radiation dose expressed in Gy for IMRT and Gy (RBE) for proton therapy

**Table 4:** Univariate analysis of patient and treatment factors and toxicity endpoints at each time point

	G-tube dependent		Median change EMD			EMD > baseline			Median % weight loss			>10 % weight loss		
Variable	Comp	3 mo	Comp	1 mo	3 mo	Comp	1 mo	3 mo	Comp	1 mo	3 mo	Comp	1 mo	3 mo
Age	0.65	0.86	0.59	0.76	0.31	0.26	0.64	0.68	0.31	0.89	0.92	0.06	0.42	0.67
Gender	>0.99	0.73	0.80	0.37	0.19	0.75	>0.99	0.72	0.65	0.84	0.42	0.72	0.75	0.19
Tumor site (NPx vs nasal/paranasal)	**0.003**	0.15	0.08	0.33	0.96	0.10	0.52	>0.99	0.17	0.19	0.29	>0.99	0.11	0.05
Histology (SCC vs non-SCC)	0.20	0.17	**0.037**	0.17	0.44	0.05	0.11	0.71	0.16	0.16	0.27	>0.99	0.20	0.34
T stage (T4 vs other)	0.52	0.30	0.051	0.19	0.71	**0.007**	0.11	0.44	0.18	0.13	0.21	0.12	0.053	**0.025**
N stage (N0 vs other)	0.21	0.48	0.17	**0.024**	0.19	0.33	0.75	>0.99	0.14	0.43	0.64	0.45	0.20	0.33
KPS (≥90 % vs <90 %)	0.06	**0.04**	0.44	0.40	0.49	0.20	0.34	0.05	0.33	0.27	0.68	>0.99	0.52	0.34
Chemotherapy (concurrent vs other)	**0.004**	0.08	**0.033**	0.15	0.38	**0.042**	0.05	0.66	**0.007**	0.09	0.96	0.66	0.11	0.11
Smoking history (none vs other)	0.11	0.08	**0.014**	0.33	0.85	**0.027**	0.20	0.27	0.05	0.16	0.36	>0.99	0.34	0.05
Active smoking at time of RT	>0.99	0.66	0.20	0.34	0.78	0.44	0.43	0.35	0.13	0.78	0.83	0.35	0.70	0.69
Diabetes mellitus	0.60	0.18	0.25	0.81	>0.99	0.26	0.58	0.55	>0.99	0.63	0.63	0.55	0.57	0.55
Modality (proton vs IMRT neck)	**<0.001**	**0.004**	**<0.001**	0.12	0.49	**<0.001**	**0.046**	0.12	0.07	0.13	0.15	0.45	0.09	**0.020**

*EMD* equivalent morphine dose, *Comp* at completion, *NPx* nasopharynx, *SCC* squamous cell carcinoma, *KPS* Karnofsky Performance Status, *RT* radiation therapy, *IMRT* intensity modulated radiation therapy *P* values <0.05 are in bold.

Table 5 reviews multivariable analysis outcomes by exact logistic regression for binary toxicity outcomes in a model including treatment modality and confounding variables of concurrent chemotherapy and the presence of cervical nodal disease. Both treatment modality and concurrent chemotherapy had a statistically significant association with g-tube dependence at the completion of radiation, while only treatment modality had an association with g-tube dependence at 3 months after radiotherapy. Only treatment modality had a statistically significant association with EMD use greater than baseline at completion of treatment. There were no variables with a statistically significant association with EMD greater than baseline at 1 and 3 months after radiation. There were no variables with a statistically significant association with percent weight loss at completion of radiation, or at 1 or 3 months after radiation.

**Table 5:** Multivariable analysis of binary outcomes

Outcome	Model predictors	Level	Odds ratio (95 % CI)	*P*
G-tube dependent at completion of RT	RT Modality	proton vs IMRT	0.03 (<0.01–0.15)	**<0.001**
	Nodal status	N0 vs N+	0.58 (<0.01–9.07)	>0.99
	Concurrent chemo	yes vs no	12.4 (1.74– > 9999)	**0.033**
G-tube dependent 1 month after RT	RT Modality	proton vs IMRT	0.11 (<0.01–0.61)	**0.028**
	Nodal status	N0 vs N+	0.88 (0.11–6.78)	>0.99
	Concurrent chemo	yes vs no	2.97 (0.42– > 9999)	0.375
EMD > baseline at completion of RT	RT Modality	proton vs IMRT	0.09 (0.01–0.57)	**0.006**
	Nodal status	N0 vs N+	0.92 (0.11–6.07)	>0.99
	Concurrent chemo	yes vs no	4.2 (0.39–66.1)	0.375

*CI* confidence interval, *G-tube* gastrostomy tube, *RT* radiation therapy, *IMRT* intensity modulated radiation therapy, *chemo* chemotherapy *P* values <0.05 are in bold.

In a subgroup analysis of the 32 patients receiving concurrent chemotherapy, there was a statistically significant correlation with a greater opioid pain medication requirement at the completion of radiation and both increasing mean dose to the oral cavity (Spearman’s ρ = 0.502, *P* = 0.003) and increasing mean dose to the esophagus (ρ = 0.361, *P* = 0.042). There was a positive correlation between increasing percent weight loss at the completion of treatment and increased opioid pain medication requirement (ρ = 0.419, *P* = 0.017).

## Discussion

Acute toxicity during head and neck radiotherapy remains significant despite advances in radiation treatment planning and delivery [1, 3]. Based on improvements in radiation dosimetry compared to IMRT that are anticipated to translated into reduced clinical toxicities, proton therapy has been proposed as a treatment modality for head and neck radiation [18], although the cost of proton therapy is generally greater than that of IMRT; approximately 2.4 times the cost of IMRT [19]. Because proton therapy is more resource-intensive and more limited in availability than photon-based treatments, additional clinical data are needed to quantify the potential benefit of proton therapy in head and neck treatments. We hope that our initial experience and data may serve in hypothesis formation for further investigation.

**Fig. 2:**
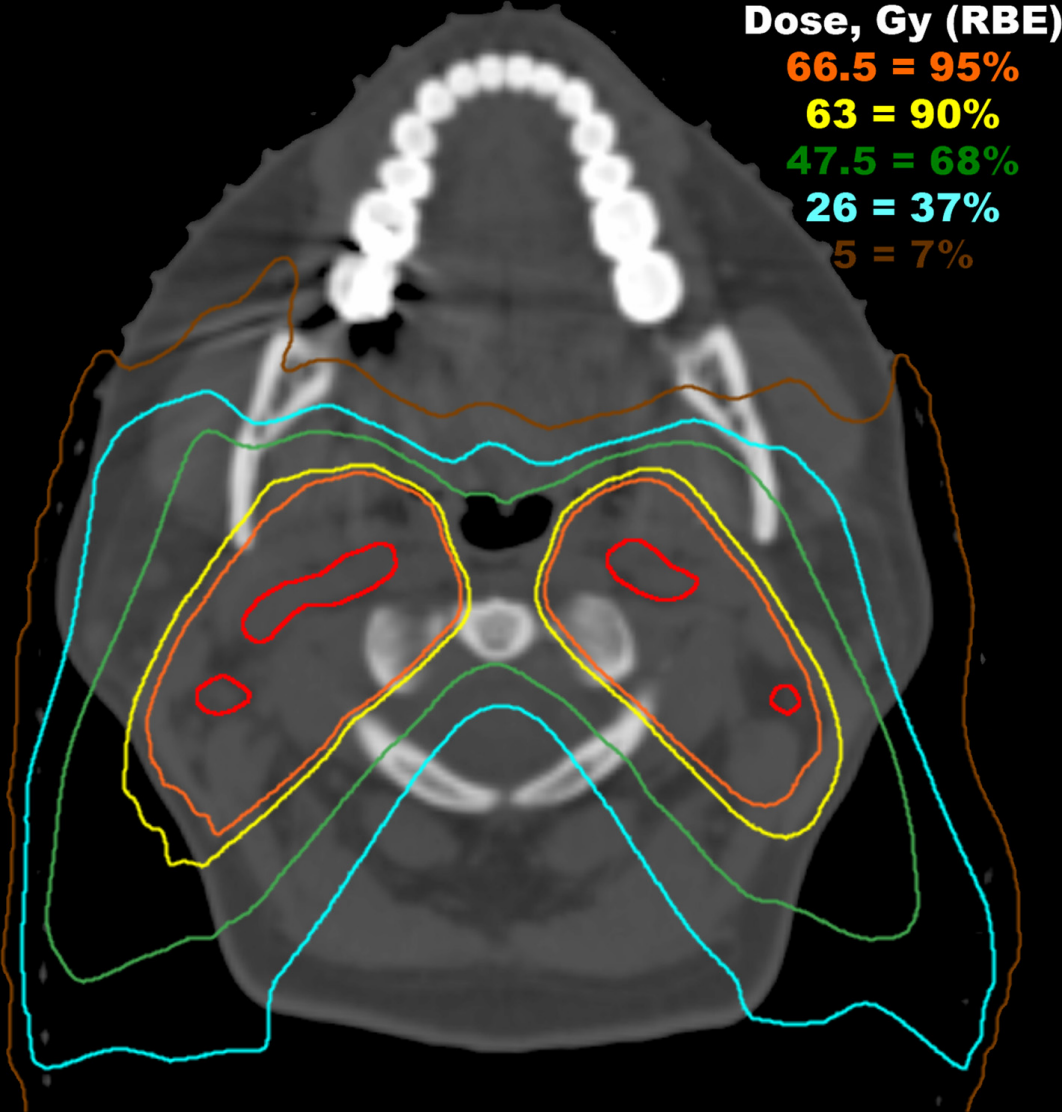
An example of a patient irradiated with bilateral involved cervical nodes. The involved lymph nodes are contoured in red. The prescription dose to the gross nodal disease was 70 Gy (RBE) in 35 fractions. Using a combination of posterior-anterior and posterior oblique fields, significant oral cavity sparing was achieved by virtue of the finite range of each proton beam

Oral mucositis in particular is an often severe [20] and costly [21, 22] toxicity of treatment associated with poor pain control despite near universal opioid pain requirements [20, 23]. Although chemotherapy is an independent risk factor for mucositis, radiation dose to the oral cavity is associated with the incidence and severity of mucositis [6], xerostomia [24], and long-term dysphagia [25], and reducing or avoiding radiation exposure to the mucosa would seem the most direct means of reducing or avoiding mucositis. In our proton cohort, significant sparing of the oral cavity was achieved, with a mean dose of 5.1 Gy (RBE) in node negative patients and 8.5 Gy (RBE) in node positive patients. Figure 2 shows an example of oral cavity sparing in a patient irradiated with involved nodal disease. Similar oral cavity sparing and mean oral cavity doses have been reported by others using spot-scanning proton therapy in comparative treatment planning for patients receiving ipsilateral head-and-neck radiation [26] and in clinical results of multi-field optimized intensity modulated proton therapy in nasopharyngeal cancer [27]. The observations of our proton cohort are in line with a retrospective study of proton therapy versus mixed photon-electron radiotherapy for pediatric salivary tumors, which found proton therapy was associated with reduced radiation dose to the oral cavity and reduced incidence of grade 2–3 oral mucositis [28].

Opioid pain medication is often required for symptomatic management of head and neck cancer patients receiving radiation. The overall opioid pain requirements of our IMRT cohort are consistent with the opioid pain utilization rates reported elsewhere in the literature [20, 23], in contrast to the markedly lower rate of change in opioid requirements in our proton cohort by the completion of treatment and at 1 month post radiation. While increased opioid requirements in response to acute toxicities are temporary in most patients (reflected by a majority of patients returning to baseline EMD by 3 months post radiation), opioids are associated with many adverse effects including somnolence, nausea, dry mouth, anorexia, and constipation [29], as well as drug interactions and risk of overdose and death [30]. Opioid-related adverse effects and events are associated with significant economic costs [31] in addition to the cost of the medications themselves and out-of-pocket expenses, which can pose a significant economic burden to patients [32].

Our institutional approach has been to recommend prophylactic g-tube placement for patients receiving IMRT, but we instituted a reactive approach for those receiving comprehensive proton therapy, none of whom required placement of a gastrostomy tube during proton therapy. Although there was a statistically significant difference in the rate of placement of g-tube between cohorts, as shown in Table 5, this comparison is less informative because of the differing approach to g-tube placement. For this reason we chose to evaluate and compare the rate of g-tube *dependence* during and after therapy. The rate of g-tube dependence in our proton cohort was very low, with no patients being g-tube dependent at the completion of radiation or at 3 months after radiation. In contrast, the significantly higher rates of g-tube dependence seen in our IMRT cohort are in line with multi-institutional data on the rates of g-tube dependence in T3 and T4 oropharyngeal patients receiving IMRT with or without concurrent chemotherapy [33]. Our results are similar to matched case control clinical data in patients treated for nasopharynx cancers, in which intensity modulated proton therapy was associated with a reduced need for gastrostomy tube placement during radiation compared to IMRT [27].

Although weight loss during and after radiation is common in patients treated for head and neck cancers, the median weight loss and significant weight loss >10 % were both fairly high in our patients. Significant weight loss has been reported in one-third to one-half of head and neck patients receiving radiation in the era prior to IMRT [34], while 12.6 % were reported to have lost >10 % weight in a series of patients receiving mostly IMRT [35]. Conversely, median weight loss during treatment was a low 2.8 % in a retrospective series of 50 patients with stage III and IV head and neck cancers receiving chemoradiation with IMRT [36]. Several variables are associated with weight loss during radiation, but among treatment factors, the use of concurrent chemotherapy is strongly associated with an increased risk of clinically significant weight loss [35]. In a multivariable model accounting for concurrent chemotherapy and node positive disease, we found no statistical difference by treatment modality in weight loss at the completion of treatment, or at 1 or 3 months post radiation. This may reflect the successful use of gastrostomy tubes to maintain weight or limit weight loss. However, the placement of g-tubes is associated with additional risks and costs, with a reported major complication rate of 7–10 % [37, 38]. As others have reported, when considering the “true” cost of different technologies over the full care cycle, including the potential reductions in costs related to reduced g-tube placement, attendant hospitalization, supplemental nutrition, opioid pain medication and associated complications, proton therapy can be cost competitive or provide higher-value care compared to IMRT [39]. Additional clinical data are needed to build more robust models of the care cycle costs of competing radiation modalities that incorporate the costs related to treatment toxicities and failures in head and neck cancer.

Our data analysis is a retrospective comparison of patients who were not randomized to treatment modality. In addition to unidentified or unknown potential selection biases inherent in retrospective analysis, as may be expected from a small cohort, there were imbalances between cohorts in patient characteristics and treatment parameters, which may be associated with toxicities. The limited sample size limits the statistical power to assess for or control all potential variables that may be associated with toxicities. Although one could speculate that the IMRT plans could have been further optimized, the mean dose to the larynx and esophagus in the IMRT cohort readily met the constraints recommended in the Radiation Therapy Oncology Group 0615 phase II trial in nasopharynx cancer [40], the oral cavity mean dose fell close to (node positive) or below (node negative) proposed oral cavity constraints [24], and the “better” spared parotid gland mean dose with IMRT was close to a standard mean dose objective [41]. The magnitude of dose sparing to OARs with proton therapy in comparison to IMRT in our cohorts reflects “real-world” gains in patients treated in an academic healthcare setting, but *in-silico* treatment planning comparisons of idealized dose distributions have also demonstrated proton therapy OAR sparing relative to IMRT in head and neck cancer [15, 26].

## Conclusions

In a multivariable model including confounding variables of concurrent chemotherapy and involved nodal disease, proton therapy was associated with a lower opioid pain requirement at the completion of radiation and a lower rate of gastrostomy tube dependence by the completion of radiation therapy and at 3 months after radiation. Compared to IMRT, comprehensive head and neck radiation therapy with proton therapy for nasopharynx, nasal and paranasal sinus tumors was associated with significantly lower mean doses to the oral cavity, esophagus, larynx, and parotid glands in both node negative patients and those with involved nodal disease.

Acknowledging the limitations of this study, our findings, together with those of other studies [27, 28], of improved oral cavity sparing, a reduced rate of g-tube dependence, and reduced opioid pain medication requirements using proton therapy merit further evaluation in a larger study with more uniform patient and treatment characteristics, and inclusion of patient-reported toxicity outcomes and quality of life endpoints.

## References

[1] Trotti A, Bellm LA, Epstein JB, Frame D, Fuchs HJ, Gwede CK (2003). Mucositis incidence, severity and associated outcomes in patients with head and neck cancer receiving radiotherapy with or without chemotherapy: a systematic literature review. Radiother Oncol.

[2] Dijkema T, Raaijmakers CP, Ten Haken RK, Roesink JM, Braam PM, Houweling AC (2010). Parotid gland function after radiotherapy: the combined michigan and utrecht experience. Int J Radiat Oncol Biol Phys.

[3] Ling DC, Kabolizadeh P, Heron DE, Ohr JP, Wang H, Johnson J (2015). Incidence of hospitalization in patients with head and neck cancer treated with intensity-modulated radiation therapy. Head Neck.

[4] Nutting CM, Morden JP, Harrington KJ, Urbano TG, Bhide SA, Clark C (2011). Parotid-sparing intensity modulated versus conventional radiotherapy in head and neck cancer (PARSPORT): a phase 3 multicentre randomised controlled trial. Lancet Oncol.

[5] Kumar R, Madanikia S, Starmer H, Yang W, Murano E, Alcorn S (2014). Radiation dose to the floor of mouth muscles predicts swallowing complications following chemoradiation in oropharyngeal squamous cell carcinoma. Oral Oncol.

[6] Bhide SA, Gulliford S, Schick U, Miah A, Zaidi S, Newbold K (2012). Dose-response analysis of acute oral mucositis and pharyngeal dysphagia in patients receiving induction chemotherapy followed by concomitant chemo-IMRT for head and neck cancer. Radiother Oncol.

[7] Eisbruch A, Kim HM, Feng FY, Lyden TH, Haxer MJ, Feng M (2011). Chemo-IMRT of oropharyngeal cancer aiming to reduce dysphagia: swallowing organs late complication probabilities and dosimetric correlates. Int J Radiat Oncol Biol Phys.

[8] Dirix P, Nuyts S (2010). Evidence-based organ-sparing radiotherapy in head and neck cancer. Lancet Oncol.

[9] Kocak-Uzel E, Gunn GB, Colen RR, Kantor ME, Mohamed AS, Schoultz-Henley S (2014). Beam path toxicity in candidate organs-at-risk: assessment of radiation emetogenesis for patients receiving head and neck intensity modulated radiotherapy. Radiother Oncol.

[10] Schulz-Ertner D, Tsujii H (2007). Particle radiation therapy using proton and heavier ion beams. J Clin Oncol.

[11] Foote RL, Stafford SL, Petersen IA, Pulido JS, Clarke MJ, Schild SE (2012). The clinical case for proton beam therapy. Radiat Oncol.

[12] Fitzek MM, Thornton AF, Varvares M, Ancukiewicz M, McIntyre J, Adams J (2002). Neuroendocrine tumors of the sinonasal tract. Results of a prospective study incorporating chemotherapy, surgery, and combined proton-photon radiotherapy. Cancer.

[13] Herr MW, Sethi RK, Meier JC, Chambers KJ, Remenschneider A, Chan A (2014). Esthesioneuroblastoma: an update on the Massachusetts Eye and Ear Infirmary and Massachusetts General Hospital experience with craniofacial resection, proton beam radiation, and chemotherapy. J Neurol Surg B Skull Base.

[14] Fukumitsu N, Okumura T, Mizumoto M, Oshiro Y, Hashimoto T, Kanemoto A (2012). Outcome of T4 (International Union Against Cancer Staging System, 7th edition) or recurrent nasal cavity and paranasal sinus carcinoma treated with proton beam. Int J Radiat Oncol Biol Phys.

[15] McDonald MW, Walter AS, Hoene TA. Technique for comprehensive head and neck irradiation using 3-dimensional conformal proton therapy. Med Dosim. 2015. 10.1016/j.meddos.2015.04.004 26002120

[16] Washington State Agency Medical Directors' Group. Interagency Guideline on Prescribing Opioids for Pain, 3rd edition, 2015. Opiod dose calculator, available online at: http://agencymeddirectors.wa.gov/mobile.html.

[17] Sohrabi S, Drabick JJ, Crist H, Goldenberg D, Sheehan JM, Mackley HB (2011). Neoadjuvant concurrent chemoradiation for advanced esthesioneuroblastoma: a case series and review of the literature. J Clin Oncol.

[18] Holliday EB, Frank SJ (2014). Proton radiation therapy for head and neck cancer: a review of the clinical experience to date. Int J Radiat Oncol Biol Phys.

[19] Goitein M, Jermann M (2003). The relative costs of proton and X-ray radiation therapy. Clin Oncol (R Coll Radiol).

[20] Elting LS, Keefe DM, Sonis ST, Garden AS, Spijkervet FK, Barasch A (2008). Patient-reported measurements of oral mucositis in head and neck cancer patients treated with radiotherapy with or without chemotherapy: demonstration of increased frequency, severity, resistance to palliation, and impact on quality of life. Cancer.

[21] Peterman A, Cella D, Glandon G, Dobrez D, Yount S (2001). Mucositis in head and neck cancer: economic and quality-of-life outcomes. J Natl Cancer Inst Monogr.

[22] Elting LS, Cooksley C, Chambers M, Cantor SB, Manzullo E, Rubenstein EB (2003). The burdens of cancer therapy. Clinical and economic outcomes of chemotherapy-induced mucositis. Cancer.

[23] Murphy BA, Beaumont JL, Isitt J, Garden AS, Gwede CK, Trotti AM (2009). Mucositis-related morbidity and resource utilization in head and neck cancer patients receiving radiation therapy with or without chemotherapy. J Pain Symptom Manage.

[24] Little M, Schipper M, Feng FY, Vineberg K, Cornwall C, Murdoch-Kinch CA (2012). Reducing xerostomia after chemo-IMRT for head-and-neck cancer: beyond sparing the parotid glands. Int J Radiat Oncol Biol Phys.

[25] Schwartz DL, Hutcheson K, Barringer D, Tucker SL, Kies M, Holsinger FC (2010). Candidate dosimetric predictors of long-term swallowing dysfunction after oropharyngeal intensity-modulated radiotherapy. Int J Radiat Oncol Biol Phys.

[26] Kandula S, Zhu X, Garden AS, Gillin M, Rosenthal DI, Ang KK (2013). Spot-scanning beam proton therapy vs intensity-modulated radiation therapy for ipsilateral head and neck malignancies: a treatment planning comparison. Med Dosim.

[27] Holliday EB, Garden AS, Rosenthal DI, Fuller CD, Morrison WH, Gunn GB (2015). Proton therapy reduces treatment-related toxicities for patients wtih nasopharyngeal cancer: A case-match control study of intensity-modulated proton therapy and intensity-modulated photon therapy. Int J Particle Ther.

[28] Grant SR, Grosshans DR, Bilton SD, Garcia JA, Amin M, Chambers MS (2015). Proton versus conventional radiotherapy for pediatric salivary gland tumors: Acute toxicity and dosimetric characteristics. Radiother Oncol.

[29] Oosten AW, Oldenmenger WH, Mathijssen RH, van der Rijt CC (2015). A systematic review of prospective studies reporting adverse events of commonly used opioids for cancer-related pain: a call for the use of standardized outcome measures. J Pain.

[30] Cheatle MD (2015). Prescription opioid misuse, abuse, morbidity, and mortality: balancing effective pain management and safety. Pain Med.

[31] Kane-Gill SL, Rubin EC, Smithburger PL, Buckley MS, Dasta JF (2014). The cost of opioid-related adverse drug events. J Pain Palliat Care Pharmacother.

[32] Craig BM, Strassels SA (2010). Out-of-pocket prices of opioid analgesics in the United States, 1999–2004. Pain Med.

[33] Setton J, Lee NY, Riaz N, Huang SH, Waldron J, O'Sullivan B (2015). A multi-institution pooled analysis of gastrostomy tube dependence in patients with oropharyngeal cancer treated with definitive intensity-modulated radiotherapy. Cancer.

[34] Beaver ME, Matheny KE, Roberts DB, Myers JN (2001). Predictors of weight loss during radiation therapy. Otolaryngol Head Neck Surg.

[35] Mallick I, Gupta SK, Ray R, Sinha T, Sinha S, Achari R (2013). Predictors of weight loss during conformal radiotherapy for head and neck cancers - how important are planning target volumes?. Clin Oncol (R Coll Radiol).

[36] Wiggenraad RG, Flierman L, Goossens A, Brand R, Verschuur HP, Croll GA (2007). Prophylactic gastrostomy placement and early tube feeding may limit loss of weight during chemoradiotherapy for advanced head and neck cancer, a preliminary study. Clin Otolaryngol.

[37] Baschnagel AM, Yadav S, Marina O, Parzuchowski A, Lanni TB, Warner JN (2014). Toxicities and costs of placing prophylactic and reactive percutaneous gastrostomy tubes in patients with locally advanced head and neck cancers treated with chemoradiotherapy. Head Neck.

[38] Grant DG, Bradley PT, Pothier DD, Bailey D, Caldera S, Baldwin DL (2009). Complications following gastrostomy tube insertion in patients with head and neck cancer: a prospective multi-institution study, systematic review and meta-analysis. Clin Otolaryngol.

[39] Thaker NG, Frank SJ, Feeley TW (2015). Comparative costs of advanced proton and photon radiation therapies: lessons from time-driven activity-based costing in head and neck cancer. J Comp Eff Res.

[40] Lee NY, Zhang Q, Pfister DG, Kim J, Garden AS, Mechalakos J (2012). Addition of bevacizumab to standard chemoradiation for locoregionally advanced nasopharyngeal carcinoma (RTOG 0615): a phase 2 multi-institutional trial. Lancet Oncol.

[41] Eisbruch A, Ten Haken RK, Kim HM, Marsh LH, Ship JA (1999). Dose, volume, and function relationships in parotid salivary glands following conformal and intensity-modulated irradiation of head and neck cancer. Int J Radiat Oncol Biol Phys.

